# Assessment of SnFe_2_O_4_ Nanoparticles for Potential Application in Theranostics: Synthesis, Characterization, In Vitro, and In Vivo Toxicity

**DOI:** 10.3390/ma14040825

**Published:** 2021-02-09

**Authors:** Saman Sargazi, Mohammad Reza Hajinezhad, Abbas Rahdar, Muhammad Nadeem Zafar, Aneesa Awan, Francesco Baino

**Affiliations:** 1 Cellular and Molecular Research Center, Resistant Tuberculosis Institute, Zahedan University of Medical Sciences, Zahedan 98167-43463, Iran; sgz.biomed@gmail.com; 2 Basic Veterinary Science Department, Veterinary Medicine Faculty, University of Zabol, Zabol 98613-35856, Iran; hajinezhad@gmail.com; 3 Department of Physics, University of Zabol, Zabol 98613-35856, Iran; 4 Department of Chemistry, University of Gujrat, Gujrat 50700, Pakistan; awananeesa90@gmail.com; 5 Institute of Materials Physics and Engineering, Department of Applied Science and Technology, Politecnico di Torino, 10129 Torino, Italy

**Keywords:** nanoparticles, tin ferrite, nanoceramics, cytotoxicity, in vivo toxicity, growth inhibition, nanomedicine

## Abstract

In this research, tin ferrite (SnFe_2_O_4_) NPs were synthesized via hydrothermal route using ferric chloride and tin chloride as precursors and were then characterized in terms of morphology and structure using Fourier-transform infrared spectroscopy (FTIR), Ultraviolet–visible spectroscopy (UV-Vis), X-ray power diffraction (XRD), Scanning electron microscopy (SEM), Transmission electron microscopy (TEM), and Brunauer–Emmett–Teller (BET) method. The obtained UV-Vis spectra was used to measure band gap energy of as-prepared SnFe_2_O_4_ NPs. XRD confirmed the spinel structure of NPs, while SEM and TEM analyses disclosed the size of NPs in the range of 15–50 nm and revealed the spherical shape of NPs. Moreover, energy dispersive X-ray spectroscopy (EDS) and BET analysis was carried out to estimate elemental composition and specific surface area, respectively. In vitro cytotoxicity of the synthesized NPs were studied on normal (HUVEC, HEK293) and cancerous (A549) human cell lines. HUVEC cells were resistant to SnFe_2_O_4_ NPs; while a significant decrease in the viability of HEK293 cells was observed when treated with higher concentrations of SnFe_2_O_4_ NPs. Furthermore, SnFe_2_O_4_ NPs induced dramatic cytotoxicity against A549 cells. For in vivo study, rats received SnFe_2_O_4_ NPs at dosages of 0, 0.1, 1, and 10 mg/kg. The 10 mg/kg dose increased serum blood urea nitrogen and creatinine compared to the controls (P < 0.05). The pathology showed necrosis in the liver, heart, and lungs, and the greatest damages were related to the kidneys. Overall, the in vivo and in vitro experiments showed that SnFe_2_O_4_ NPs at high doses had toxic effects on lung, liver and kidney cells without inducing toxicity to HUVECs. Further studies are warranted to fully elucidate the side effects of SnFe_2_O_4_ NPs for their application in theranostics.

## 1. Introduction

Today, nanomedicine deals with the application of accurately engineered materials to emerge new modalities in theranostics [1]. These nanomaterials are ultra-small and highly reactive, making useful tools for overcoming limitations observed in conventional theranostic agents [2]. Cancer cells have high hydrogen peroxide levels that can catalyze the Fenton reaction, resulting in anticancer reactive oxygen species (ROS) [3]. Ferrite nanoparticles are of huge interest due to their comprehensive range of usages in many fields such as high-technology devices, catalysis, medicine, removal of organic contaminants, and pharmaceutical science [4,5]. Spinel ferrites are the foremost class of such materials because of their intensified catalytic, optical, electronic, magnetic, and electrical characteristics. Spinel ferrites possess generalized formula of M^II^Fe_2_O_4_ (where M^II^ = Fe, Mn, Ni, etc.), and the unit cell with 32 oxygen atoms possess face-centred compact packing. By varying the nature of the bivalent ion M^2+^, one can get ferrites with different magnetic and physical properties [6,7].

It has been established that SnFe_2_O_4_ nanocrystals facilitate the Fenton reaction within cancer cells and induce apoptotic cell death. However, they seem not to affect cells with normal physiology. Thus, SnFe_2_O_4_ nanocrystals could serve as selective cancer eradicators [8]. Metal oxide-based nanoparticles have been making notable endowments to promote novel delivery systems in chemotherapy and radiation therapy. The application of inorganic nanoparticles for cancer treatment and drug delivery is growing day by day because of the numerous experiments conducted to increase their bioavailability and effectiveness [9]. Metal oxide-based nanoparticles are a group of inorganic materials that have attracted considerable attention in nanomedicine and biotechnology [10]. To the best of our knowledge, there is no data in the literature regarding the short-term toxicity of SnFe_2_O_4_ nanoparticles (NPs) in laboratory animals. Few works have been conducted to explore the consequences of SnFe_2_O_4_ NPs on normal and cancer cell lines in the cell culture medium. It was noted that SnFe_2_O_4_ NPs were well tolerated by normal cells, exhibiting normal physiological levels of catalase. In contrast, SnFe_2_O_4_ NPs showed effective in vitro cytotoxicity against cancer cells by activating heterogeneous Fenton reactions—an efficacious and auspicious selective cancer cell treatment process [8].

If not killing normal cells, magnetic NPs could serve as a promising multifunctional theranostic nano-platform in imaging guided cancer therapy. Despite the diverse applications of SnFe_2_O_4_ NPs in industry and medical engineering, there is a paucity of data about the in vivo and in vitro toxicity and relevant fundamental mechanisms of these structures. To the best of our knowledge, the biochemical effects of SnFe_2_O_4_ NPs have not been investigated in laboratory rodents. Furthermore, there is no published data regarding the histopathological effects of SnFe_2_O_4_ NPs on organs such as the liver, kidney, lung, testis, and brain. The current work aims at bridging this gap and is directed to investigate the potential in vitro and in vivo toxicity of SnFe_2_O_4_ NPs on different cells/organs.

## 2. Materials and Methods

### 2.1. Materials

Ferric chloride hexahydrate (FeCl_3_^·^6H_2_O), stannous chloride dihydrate (SnCl_2_·2H_2_O), acetonitrile, and sodium hydroxide (NaOH) were purchased from Sigma Aldrich (Sigma-Aldrich, Darmstadt, Germany) and used as received.

### 2.2. Preparation of SnFe_2_O_4_ NPs

A facile and cost-effective hydrothermal approach was followed to synthesize high-quality nano-sized particles [11]. Following this typical procedure, SnFe_2_O_4_ NPs were synthesized with specific ratios of precursors, i.e., tin chloride, ferric chloride and sodium hydroxide. In the first step, ferric chloride (3.03 g) and tin chloride (0.6 g) were added in stoichiometric amounts and subjected to stirring for 30 min; then, 10 mL of 1 M sodium hydroxide was added as precipitating agent and the batch was further stirred vigorously for 90 min at 80 °C. The resultant reaction mixture was then transferred to stainless steel autoclave for 24 h at 150 °C. The as-prepared particles were collected, filtered, and washed with ethanol. The filtered product was placed in an oven at 100 °C and, after this, calcination of the prepared SnFe_2_O_4_ NPs was carried out at 300 °C in a muffle furnace for 4 h to remove any kind of impurity, if present.

### 2.3. Characterization

Diverse analytical characterization approaches were extensively utilized to explore the morphological and structural attributes of resultant samples. Ultraviolet-visible spectrum was measured through UV-vis spectrophotometer (Shimazdu Corporation, Kyoto, Japan) with resolution of 0.1 nm and accuracy of 0.3 nm. The functional group characterization was accomplished while taking FTIR spectra from spectrometer within the range between 500 and 4000 cm^−1^. X-ray diffractometer (Miniflex 600, Rigaku Corporation, Tokyo, Japan) was utilized to investigate XRD patterns that were recorded by using CuKα radiation (1.54 Å) from 25° to 70° with angle of deviation of 0.01° and accuracy of 0.005°. The morphology and composition of samples were investigated by SEM and EDS, respectively (SEM equipped with EDS, JSM-6390LV, JEOL, Tokyo, Japan); TEM analysis was performed as well (Philips EM-400, Amsterdam, Netherlands; operating voltage 120 kV). The adsorption-desorption of N_2_ at −196 °C was used for textural characterization. BET method was performed for evaluating the specific surface area of SnFe_2_O_4_ NPs; pore volume and mean pore size were determined by using the Barrett–Joyner–Halenda (BJH) method.

### 2.4. Cells and Culture Condition

A549 human lung cancer cells were selected as an in vitro solid tumor model. Human embryonic kidney cells (HEK293) and human umbilical vein endothelial cells (HUVECs) were chosen as widely studied non-cancerous cell lines for cytotoxicity assessment.

HEK293 and A549 cells were obtained from cell repository of Royan Institute (Tehran, Iran). HUVEC cells were a kind gift from Dr. Roghayeh Sheervalilou. Both HEK293 and HUVEC cell lines were maintained in Dulbecco’s Modified Eagle’s medium (Gibco, Rockville, MD, USA), while the A549 cells were cultivated in RPMI1640 medium (Gibco, Rockville, MD, USA). Culture mediums were augmented with 10% heat-inactivated fetal bovine serum (FBS, Biochrome, Berlin, Germany), 250 µg/mL amphotericin B (Sigma-Aldrich, Steinheim am Albuch, Germany), 50 U/mL penicillin (Sigma-Aldrich, Steinheim am Albuch, Germany), and 50 µg/mL streptomycin (Sigma-Aldrich, Steinheim am Albuch, Germany) and kept at standard cell culture conditions [12]. The culture media were changed every 2 or 3 days. At 80% confluency, cells attached to the culture flask were trypsinized and passed to new culture flasks (Jet biofil, Sorfa, Germany) at a cell density of 5000 cells/cm^2^.

### 2.5. In Vitro Cytotoxicity Evaluation

The growth inhibitory effect of SnFe_2_O_4_ NPs was assessed using a tetrazolium (MTT)-based colorimetric assay [13]. Cells (5 × 10^3^ cells/well) were seeded in a 96-well plate (Sorfa Life Science Research Co., Ltd., Zhejiang, China) and incubated overnight to allow cell adhesion. SnFe_2_O_4_ NPs (50 mg) were dispersed in distilled water to prepare a 169.8 mM stock solution. Cells were then treated with increasing concentrations (50–1600 μM) of SnFe_2_O_4_ NPs prepared in a culture medium. After 24 to 72 h incubation, MTT dye (Sigma-Aldrich, St. Louis, MO) at a dosage of 5 mg/mL was poured into a particular well and nurtured for 3 h at 37 °C. Optical density was assessed by eluting the dye with dimethyl sulfoxide (Sigma, St. Louis, MO, USA). The absorbance was measured at 570 nm using a SpectraMax Gemini microtiter plate reader (Molecular Devices, Sunnyvale, CA, USA). Cell growth (expressed as a percentage) was ascertained by dividing the absorbance measured for treated cells by the absorbance measured for non-treated cells. The evaluation was repeated threefold.

### 2.6. Animal Treatments and Experimental Design

In the current experimental study, thirty-two male adult white rats (Wistar breed) with a mean weight of 234 g were utilized. The animals were acquired from the animal breeding colonies present at the centre of laboratory animal research of Zabol University. Rats were housed in groups of three animals in each polycarbonate cage. The room where the animals were held had a standard temperature (25 °C) and light program. Rats had free access to normal laboratory rodent pellets (manufactured by Javaneh-Khorasan company, Mashhad, Iran). In order to get the animals ready for the experiments, rats were subjected to an adaptation period that was lasted for two weeks. The animal treatments were carried out according to the international guidelines of Care and Use of Laboratory Animals (from NIH Publication No. 85–23) and Zabol University’s institutional ethical research committee instructions. Rats were arbitrarily and equally distributed into four classes as follows: the healthy control rats received 0.5 mL physiological saline intraperitoneally for three weeks, the second group received daily intraperitoneal 0.1 mg/kg dose of SnFe_2_O_4_ NPs, the third group was treated by daily intraperitoneal injection of 1 mg/kg SnFe_2_O_4_ NPs, and the fourth group received the highest dose (10 mg/kg) of SnFe_2_O_4_ NPs intraperitoneally for twenty-one days. Finally, after three weeks, blood samples were collected by retro-orbital sinus puncture and immediately centrifuged (1500 rpm for 10 min). After being centrifuged, the obtained serum samples were stored at −20 °C till the start of the biochemical analysis.

### 2.7. Serum Biochemical Parameters

The quantities of blood urea nitrogen (BUN), aspartate aminotransferase (AST), alanine aminotransferase (ALT), and creatinine present in serum samples were determined using the Pars Azmoon Company colorimetric kits. The Selectra Pro M autoanalyzer (Vital Scientific, Spankeren, Netherlands) was used for biochemical analysis. Serum malondialdehyde content was calculated by employing the procedure reported by Ohkawa et al. [14]. After three weeks of intraperitoneal injections, rats were euthanized in a human fashion by a high dose of pentobarbital anesthesia. Liver, kidney, lung, and heart tissues were sliced in 0.5 cm × 0.5 cm × 0.5 cm cubes and maintained in neutral buffered formalin for two days. After 48 h, the formalin was replaced by new formalin, and the specimens were sent to the histopathological laboratory for paraffin treatment and paraffin block making.

Paraffin blocks were cut into 4–6 µm thick segments. Successive sections were pigmented with hematoxylin-eosin and examined by a light microscope (Olympus, Tokyo, Japan) for detecting any histopathological changes.

### 2.8. Statistical analysis

The statistical analysis of biochemical data was performed by SPSS 20 (IBM Statistics, New York, NY, USA). The one-way analysis of variance (ANOVA) and the Tukey post-hoc test was employed to detect the variation among the control group and experimental groups at P ˂ 0.05.

## 3. Results

### 3.1. Characterization of SnFe_2_O_4_ NPs

**Figure 1:**
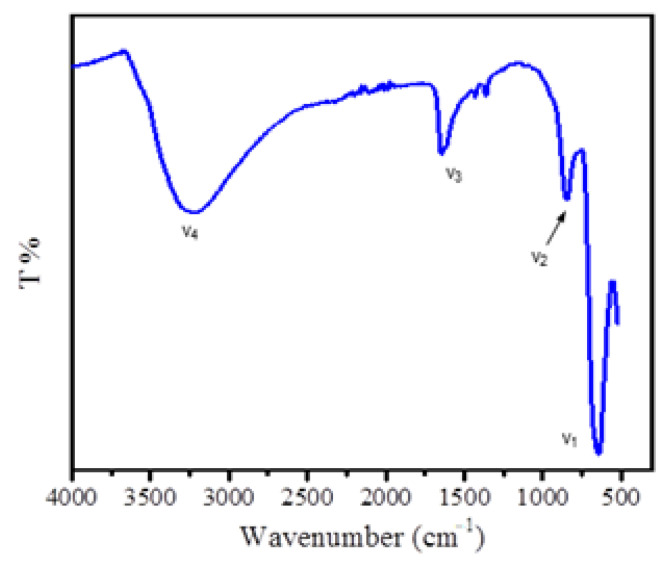
FTIR spectrum of fabricated SnFe_2_O_4_ nanoparticles (NPs).

The FTIR spectrum of as-synthesized SnFe_2_O_4_ NPs is shown in Figure 1. The characteristic absorption bands in the range between 1600 and 1644 cm^−1^ were attributed to the statistical bending vibration of O–H bond of hydroxyl group (v_3_). The intense peak at 614.25 cm^−1^ corresponds to the presence of Sn–O bond of tin-ferrite NPs (v_1_). The broad signal in the range of 3172.91–3366.55 cm^−1^ indicates the stretching of O–H bond (v_4_). Moreover, the adsorption band at 846.99 cm^−1^ corresponds to the vibrational modes of tin and iron in tetrahedral and octahedral sites (v_2_).

**Figure 2:**
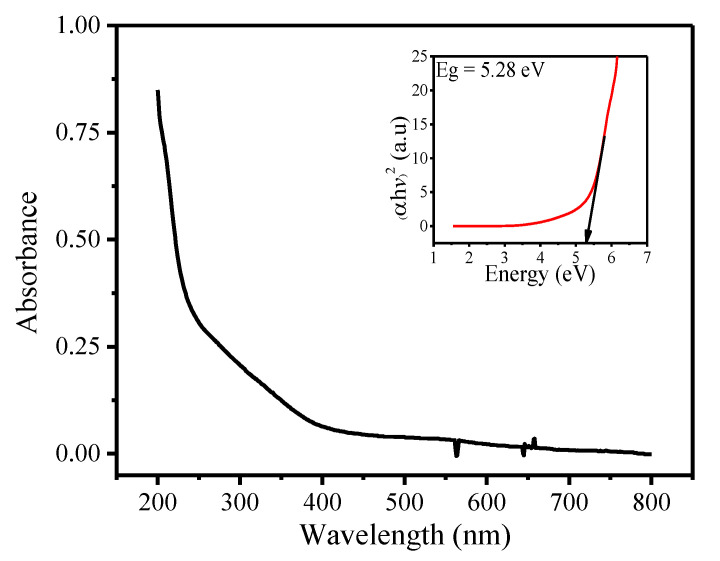
UV-visible spectrum along with Tauc plot of fabricated SnFe_2_O_4_ NPs.

Figure 2 represents the UV-Vis spectrum of SnFe_2_O_4_ NPs, which was used to calculate the value of band gap energy. The energy required to excite electrons from valence to conductance band, for this sample, is found to be 5.23 eV. This numerical value is estimated from Tauc plot. Furthermore, the high values predict the decrease in the size of nanoparticle.

**Figure 3:**
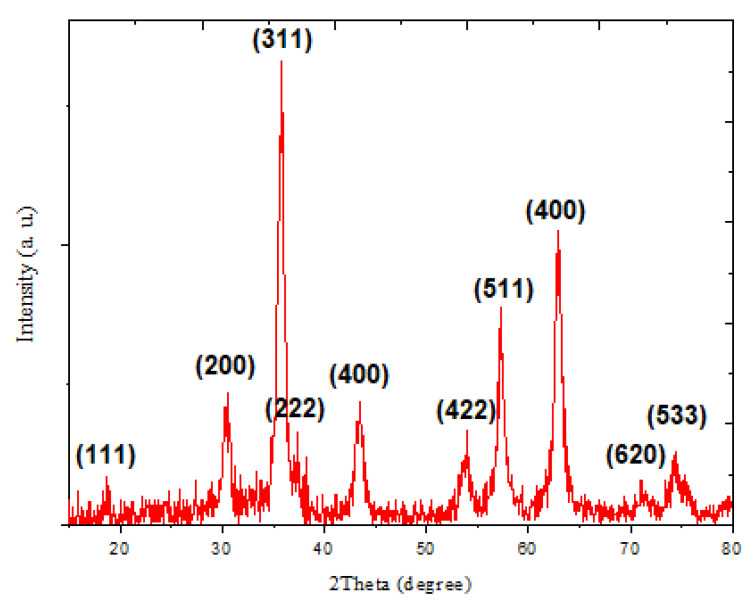
XRD pattern of SnFe_2_O_4_ NPs prepared by hydrothermal method.

XRD pattern (Figure 3) of fabricated SnFe_2_O_4_ NPs represents a series of narrow and broad diffraction signals characteristic for pure and good-quality nanoparticles. In accordance to JCPDS database, the single-phase spinel-related structure was identified (code 22-1086) [11]. The mean size of crystallites was determined by using Scherrer equation and was found in the range of 15 to 50 nm. The SnFe_2_O_4_ NPs exhibited the major diffraction signals attributed to (111), (220), (311), (222), (400), (422), (511), (440), (620), and (533) reflections, corresponding to the crystallographic planes of highly defined lattice of SnFe_2_O_4_ NPs.

**Figure 4:**
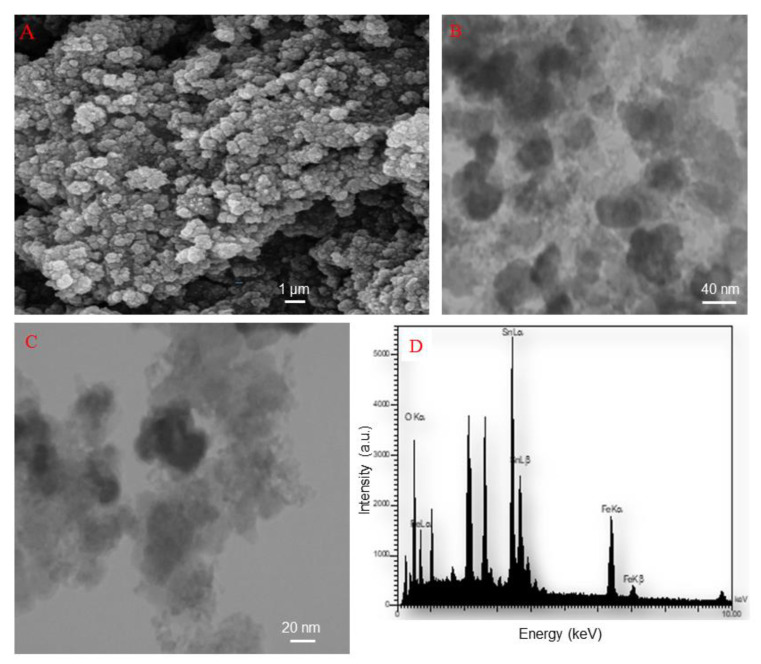
SEM image (**A**), TEM image (**B**,**C**), and EDS spectra (**D**) of synthesized SnFe_2_O_4_ NPs.

The SEM micrograph for SnFe_2_O_4_ NPs that have been produced by hydrothermal treatment is shown in Figure 4A. The particles prepared by this method are mono-dispersed and highly pure because of preferential nucleation and growth in the fabrication process, SnFe_2_O_4_ NPs with distinctive spherical morphology have been created. Moreover, the agglomeration/aggregation of NPs can also be seen in SEM image.

TEM micrographs of as-prepared SnFe_2_O_4_ NPs are reported in Figure 4B,C. The TEM micrographs reveal that SnFe_2_O_4_ NPs fabricated via hydrothermal method exhibit quite uniform particle distribution and morphology with round shape. TEM analysis disclosed the size of NPs in the range of 15–50 nm.

EDS pattern shown in Figure 4D ascertained the presence of iron, oxygen, and tin in the NPs, in accordance with theoretical expectations; this is also consistent with XRD results confirming the tin-ferrite phase (SnFe_2_O_4_).

**Figure 5:**
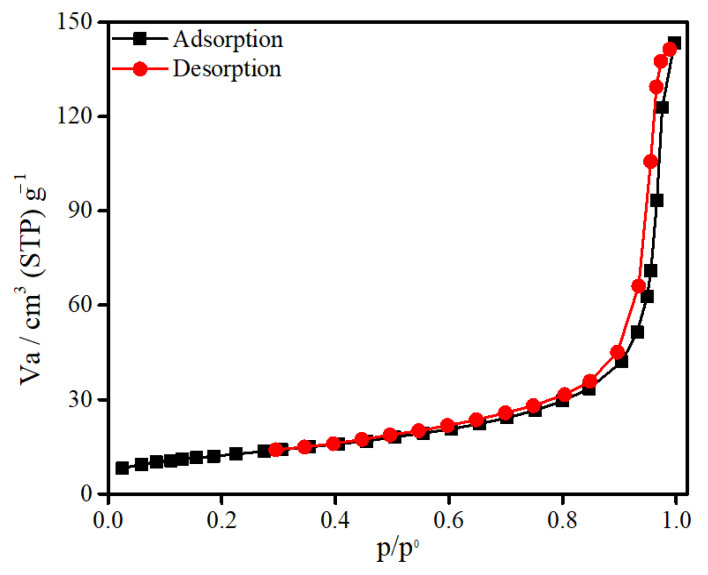
Nitrogen adsorption-desorption isotherm plot of synthesized SnFe_2_O_4_ NPs.

In order to estimate the surface area, pore volume, and pore size distribution of SnFe_2_O_4_ NPs, BET analysis was performed (Figure 5). This material demonstrates the adsorption-desorption isotherm of type-IV with the value of hysteresis loop in the range of 0.52–0.96 indicating the existence of numerous pores in the described structure of SnFe_2_O_4_ NPs. The specific surface area, pore volume and mean pore diameter of as-prepared SnFe_2_O_4_ NPs are found to be 42.92 m^2^/g, 0.212 cm^3^/g, and 19.76 nm, respectively.

### 3.2. Determination of Cell Sensitivity to SnFe_2_O_4_ NPs

**Figure 6:**
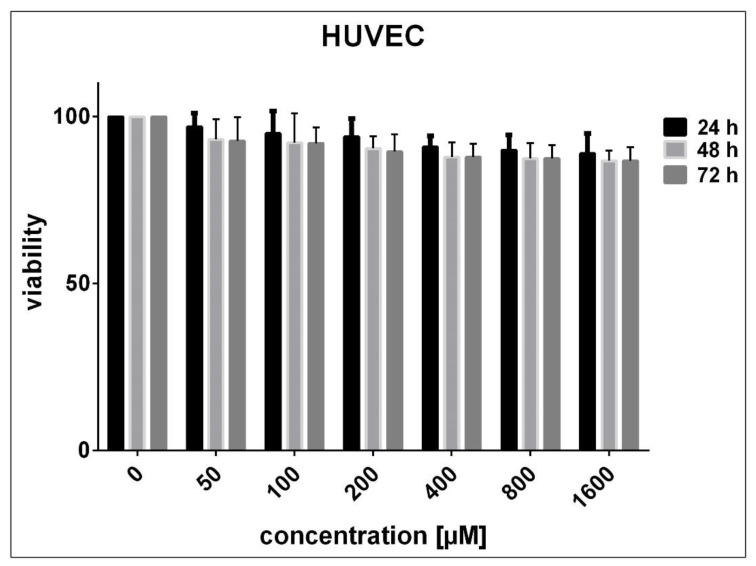
Cytotoxic effects SnFe_2_O_4_ NPs on HUVECs using MTT analysis following 24, 48, and 72 h exposure. No significant reduction in cell viability was observed (P > 0.05 compared with untreated cells).

**Figure 7:**
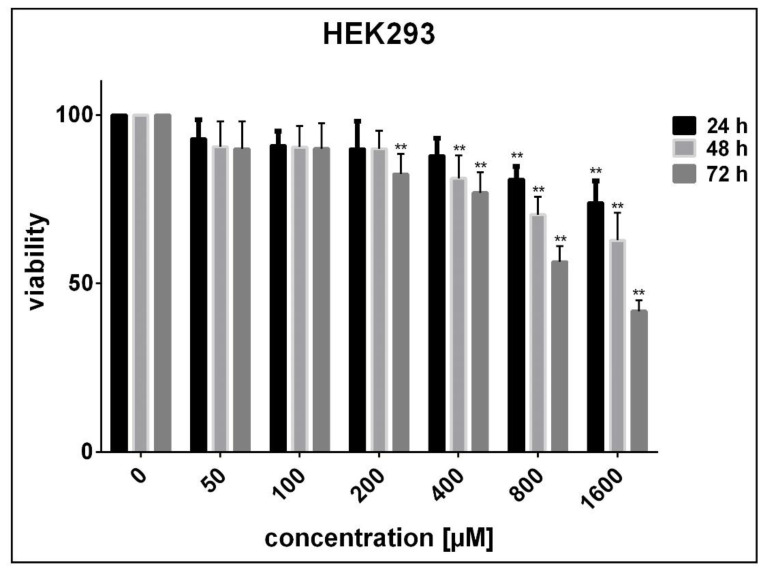
Cytotoxic effects SnFe_2_O_4_ NPs on HEK293 cells by using MTT analysis following 24, 48, and 72 h exposure. (** P < 0.05 compared with untreated cells).

**Figure 8:**
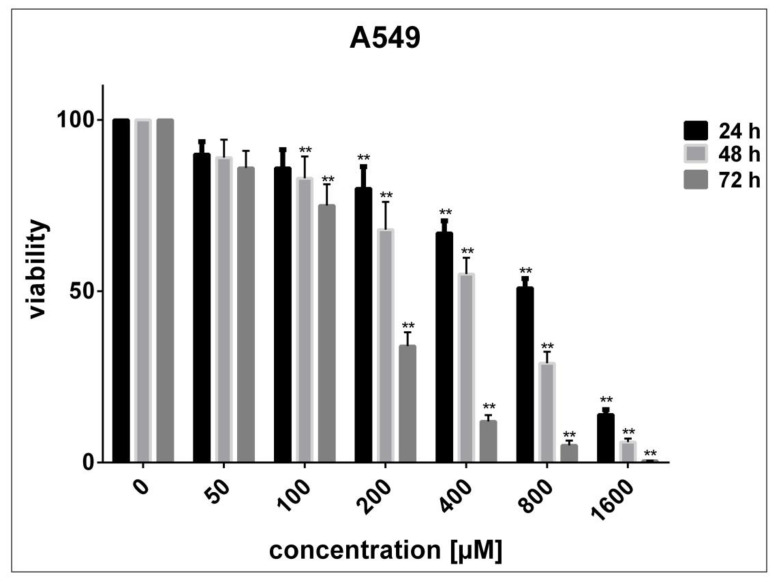
Cytotoxic consequences SnFe_2_O_4_ NPs on A549 lung cancer cells by using MTT analysis following 24, 48, and 72 h exposure. (** P < 0.05 compared with untreated cells).

No significant decrease in the viability of HUVECs was noticed after 24 to 72 h exposure to SnFe_2_O_4_ NPs (P > 0.05 compared with adjusted untreated cells) (Figure 6). On the contrary, after being exposed for 24, 48, and 72 h to high concentrations of SnFe_2_O_4_ NPs, HEK293 cells exhibited a marked decrease in viability in a concentration- and time-dependent fashion (P < 0.05 in contrast with adjusted unprocessed cells) (Figure 7). The half-maximal inhibitory concentrations (IC50) estimated using GraphPad Prism software version 7.0 for 24, 48, and 72 h treatment of HEK293 cells with SnFe_2_O_4_ NPs were 3716, 2195, and 1116 μM, respectively. As regards A549 cells, significant concentration- and time-dependent cell toxicity was observed after exposure to different concentrations of SnFe_2_O_4_ NPs, reducing the cell viability by 10 to 99.5% at a given time (P < 0.05 in contrast with adjusted unprocessed cells) (Figure 8). The calculated IC50 values for the treatment of A549 cells with SnFe_2_O_4_ NPs were 651.1 μM (after 24 h), 381.3 μM (after 48 h), and 141.2 μM (after 72 h).

### 3.3. Biochemical Results

Intraperitoneal injections of SnFe_2_O_4_ at a dose of 0.1 mg/kg had no significant effect on serum aspartate aminotransferase, serum alanine aminotransferase, serum blood urea nitrogen, and serum creatinine levels (P < 0.05) (Table 1). The intraperitoneal treatment of rats with SnFe2O4 at 4 mg/kg body weight caused no biochemical changes in serum liver enzymes and serum BUN, and creatinine levels (P < 0.05) (Table 1). The high-dose administration of SnFe_2_O_4_ caused no significant changes in serum alanine aminotransferase and serum aspartate aminotransferase; however, serum levels of blood urea nitrogen and creatinine were significantly higher compared to the control rats (P < 0.05). Serum lipid peroxidation was also increased in the high-dose treated rats (P < 0.05).

**Figure 9:**
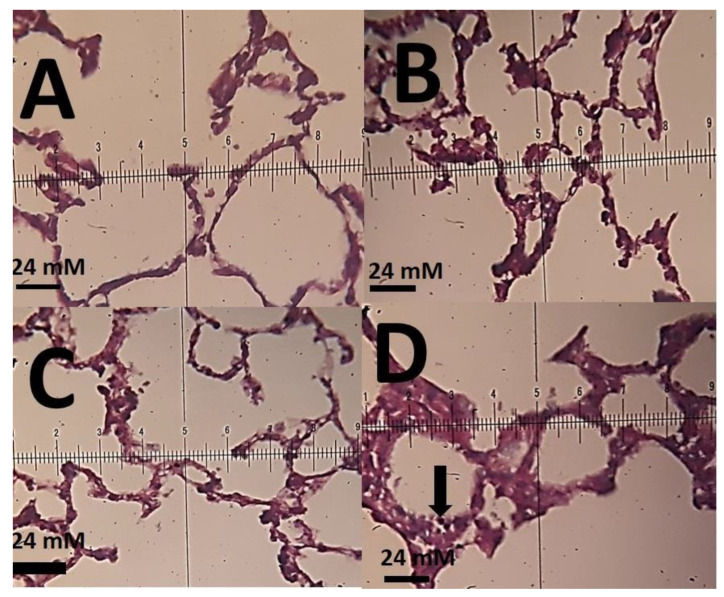
(**A**): Alcian blue micrograph of the lung of a control rat representing normal lung morphology with normal type I and type II pneumocytes (×40); (**B**): lung section of a rat treated with 0.1 mg/kg SnFe_2_O_4_ NPs. Alcian blue staining (×40); (**C**): lung section of a rat treated with SnFe_2_O_4_ NPs at a dose of 1 mg/kg. Alcian blue staining (×40); (**D**): lung section of a rat treated with SnFe_2_O_4_ NPs at a dosage of 10 mg/kg showing hypertrophy of lung alveoli (arrow), Alcian blue staining (×40).

**Figure 10:**
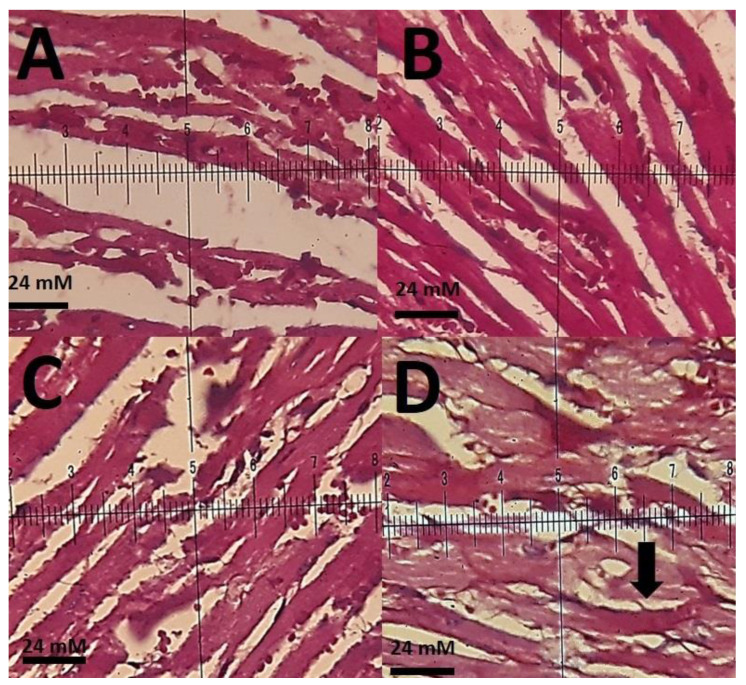
(**A**): Alcian blue staining of the heart muscle of a control rat representing normal histopathological aspects (×40); (**B**): heart section of a rat treated with SnFe_2_O_4_ NPs at a dosage of 0.1 mg/kg. Alcian blue staining (×40); (**C**): heart section of a rat treated with SnFe_2_O_4_ NPs at a dose of 1 mg/kg. Alcian blue staining (×40); (**D**): heart section of a rat treated with SnFe_2_O_4_ NPs at a dosage of 10 mg/kg. Heart muscle hypertrophy is the main feature (arrow), Alcian blue staining (×40).

**Figure 11:**
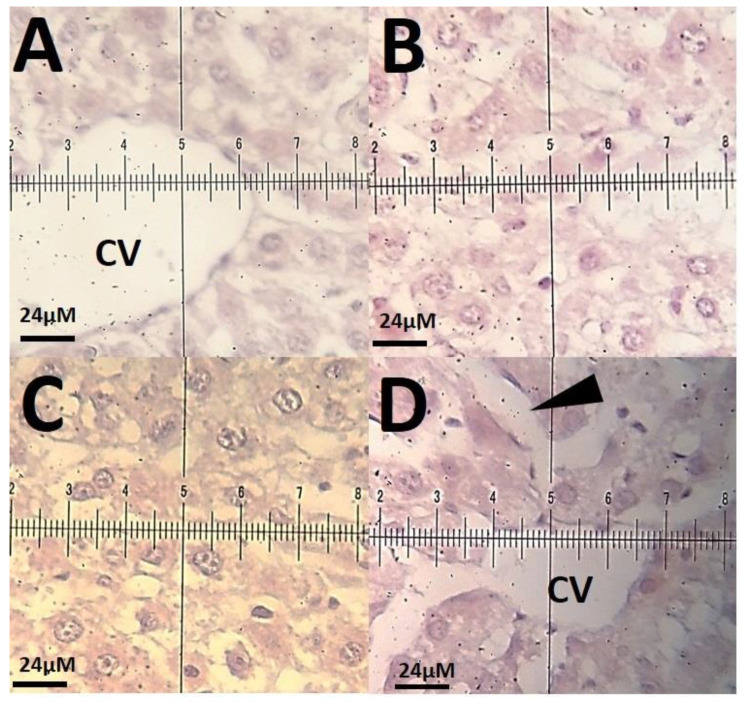
(**A**): Periodic acid-Schiff (PAS) staining of a liver section of a control rat showing the normal histology of hepatocytes and hepatic cords around the central vein (CV). Bar = 24 μm (×40); (**B**): liver segments of a rat processed with SnFe_2_O_4_ NPs at a dose of 0.1 mg/kg with no prominent histopathological change. PAS-pigmentation (×40); (**C**): liver section of a rat collected the 1 mg/kg dosage of SnFe_2_O_4_ NPs showing normal morphology of hepatocytes and hepatic cords. PAS-staining (×40); (**D**): liver segment of a rat treated with intraperitoneal injections of 10 mg/kg SnFe_2_O_4_ NPs, showing signs of disarrangement of hepatic cords (arrow point). PAS-staining (×40). Scale bar indicates 40 μm.

**Figure 12:**
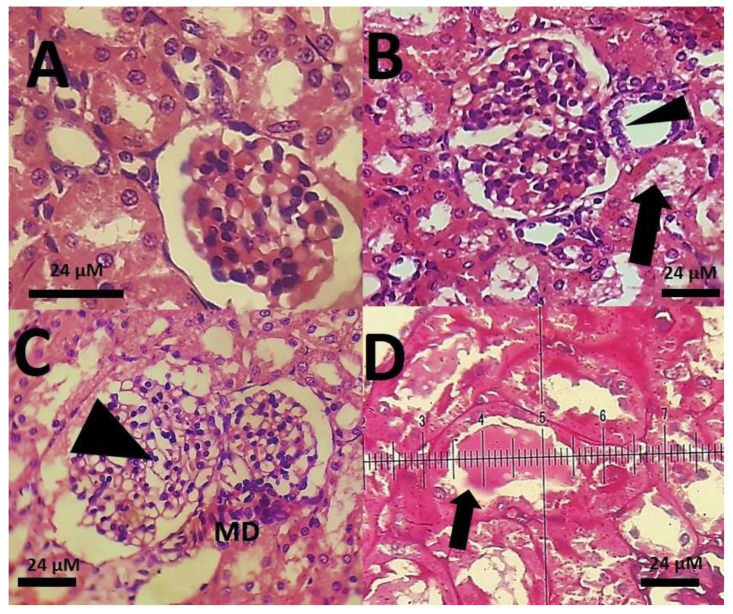
(**A**): Kidney portrayal of a rat stained with Alcian blue showing normal glomerular morphology and normal tubules; (**B**): renal image of a rat treated with SnFe_2_O_4_ NPs at a dosage of 0.1 mg/kg with normal glumerole (arrow point) and the adjacent macula densa apparatus (MD); (**C**): rat kidney processed with SnFe_2_O_4_ NPs at a dosage of 0.1 mg/kg. Macula densa and glomerules have normal structure, but the preliminary signs of inflammation and renal failure are present (arrow). (**D**): A kidney Alcian blue-stained section of a rat treated with SnFe_2_O_4_ NPs at a dosage of 10 mg/kg, hyaline casts are seen (arrow point) Alcian blue staining. (40× magnification).

**Figure 13:**
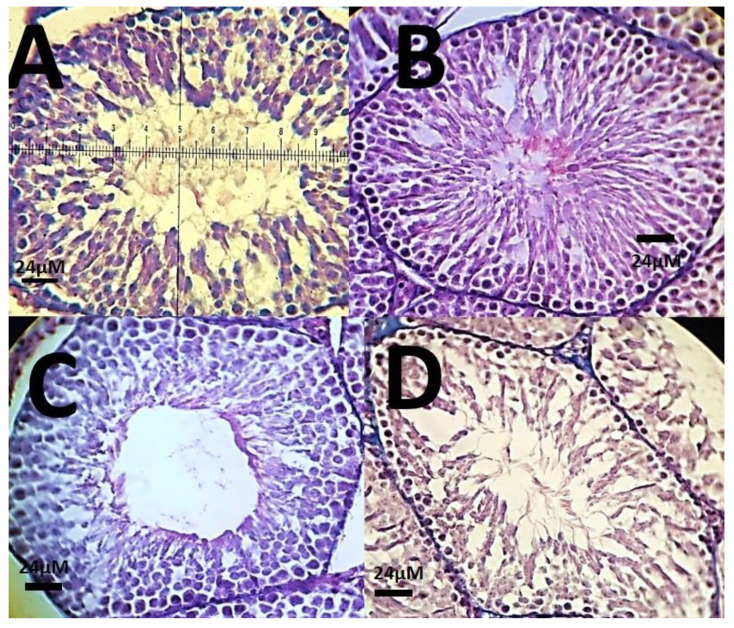
(**A**): Testis histological pattern of a rat showing normal testicular histopathology and normal seminiferous tubules through Periodic acid–Schiff (PAS) staining (×40); (**B**): PAS-stained histology section of a rat treated with SnFe_2_O_4_ NPs at a dosage of 0.1 mg/kg with normal testis appearance following periodic acid–Schiff staining; (**C**): Testis of a rat treated with SnFe_2_O_4_ NPs at a dosage of 1 mg/kg after (arrow point). (**D**): A testis PAS-stained section of a rat treated with SnFe_2_O_4_ NPs at a dosage of 10 mg/kg, no obvious sign of inflammation and necrosis is present. PAS staining (×40).

**Figure 14:**
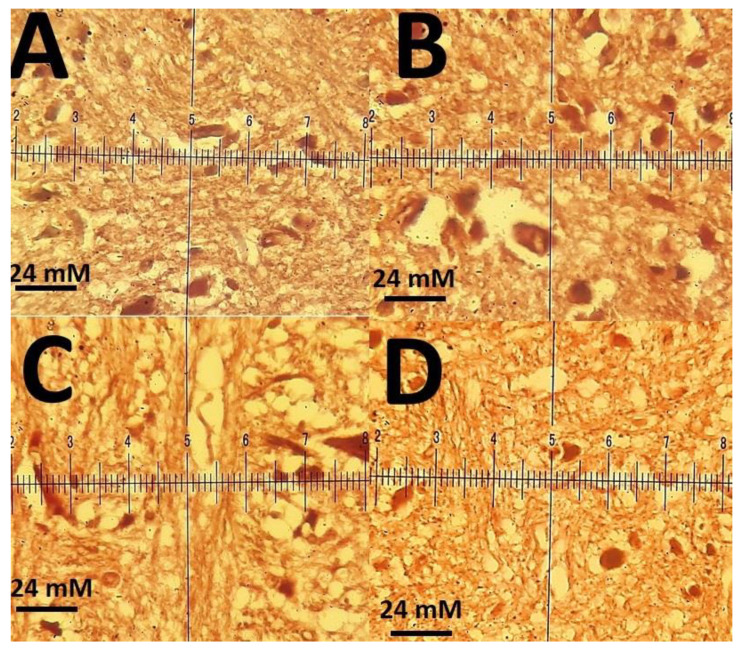
(**A**): Brain histological pattern of a rat showing normal brain histopathology. trichrome mason staining (×40); (**B**): trichrome Mason-stained histology section of a rat treated with SnFe_2_O_4_ NPs at a dosage of 0.1 mg/kg with normal brain histology following trichrome mason staining; (**C**): Brain of a rat treated with 1 mg/kg SnFe_2_O_4_ NPs (arrow point). (**D**): A brain trichrome mason-stained section of a rat treated with SnFe_2_O_4_ NPs at a dosage of 10 mg/kg, no obvious sign of inflammation and necrosis is present. PAS-staining (×40).

The histopathological investigations of different tissue samples, including lung, brain, liver, heart, kidney, and testis, are shown in Figure 9, Figure 10, Figure 11, Figure 12, Figure 13 and Figure 14. In the current investigation, various tissues were stained with specific colors. As seen in Figure 9, the Alcian blue lung section of a control rat showed normal lung structure with normal type I and type II pneumocytes (Figure 9A). The groups subjected to the 0.1 mg/kg and 1 mg/kg doses of SnFe_2_O_4_ NPs also showed normal lung histopathology (Figure 9B,C). The lung section of a rat treated with 10 mg/kg dose of SnFe_2_O_4_ NPs showed hypertrophy of lung alveoli (Figure 9D). Alcian blue staining of the heart muscle of rats treated with SnFe_2_O_4_ NPs at a dosage of 0.1 and 1 mg/kg showed normal morphology (Figure 10B,C). The heart section of a rat treated with SnFe_2_O_4_ NPs at the dosage of 10 mg/kg showed some signs of cardiac hypertrophy (Figure 10D). The histopathological examination of liver tissues showed the normal histology of liver cells and hepatic cords in liver segments of rats treated with SnFe_2_O_4_ NPs at a dose of 0.1 mg/kg (Figure 11B). Periodic acid-Schiff (PAS) staining of a liver section of rats treated with the 1 mg/kg dose of SnFe_2_O_4_ NPs showed no prominent histopathological change (Figure 11C). Liver micrographs of the high-dose treated rats showed some signs of disarrangement of hepatic cords—an indicator of early liver cell damage—around the central veins (Figure 11D).

The histopathological changes in the kidneys of rats were more severe. While the glomerular structure of rats treated with the low dose of SnFe_2_O_4_ NPs was normal (Figure 12B), the groups receiving the high doses of SnFe_2_O_4_ NPs showed preliminary signs of inflammation and renal failure; hyaline casts were also present (Figure 12D). Brain and testis tissues showed an overall normal histological appearance (Figure 13; Figure 14).

## 4. Discussion

By performing in vitro experiments, we examined the sensitivity of two non-cancerous cell lines, derived from the human umbilical vein and human embryonic kidney, towards newly synthesized SnFe_2_O_4_ NPs. We found that HUVECs were more resistant to SnFe_2_O_4_ NPs as compared with HEK293 cells. These findings suggest that the cytotoxicity of the developed SnFe_2_O_4_ NPs may be tissue-specific. In our study, in agreement with the MTT assay results, severe histopathological changes were observed in the kidneys of rats receiving SnFe_2_O_4_ NPs. On the other hand, SnFe_2_O_4_ NPs induced a marked decrease in the number of living A459 lung cancer cells. This is in agreement with the detection of Lee et al., reporting that sonicated SnFe_2_O_4_ nanocrystals profoundly enhanced ROS production via the Fenton reaction and exerted significant cytotoxic effects against lung cancer cells [8]. Moreover, they observed no marked cytotoxicity for the normal lung fibroblasts, which is consistent with our findings.

Interesting results were also reported for other metal oxide-based nanoparticles in the recent literature. Zhou and co-workers reported that treatment of A549 cells with monodispersed Fe_2_O_3_ nanoparticles did not affect cell growth and morphology [15]. Ramalingam et al. showed that Fe_2_O_3_ NPs inhibited the proliferation of PA-1 ovarian cancer cell line (IC50 = 120 µg/mL after 24 h) [16]. Zerboni and colleagues reported a significant concentration-dependent decrease in the number of viable A549 cells after 24 h and 48 h exposed to CuO NPs at concentrations of 15, 20, and 25 µg/mL. In contrast, ZnO NPs were not able to induce such cytotoxic effects at concentrations less than 25 µg/mL [17]. Li et al. showed that low concentrations of iron oxide-based magnetic NPs markedly diminished the cell viability of A549 cells and exerted noticeable synergistic effects against malignant cells [18]. Ghasemi et al. examined the effects of superparamagnetic iron oxide NPs on the viability of HEK293 normal human cells. After 24 h, they observed marked inhibitory effects started at 25 µg/mL (with 6% decrease in cell viability of HEK293 cells compared with untreated cells) and increased at 200 µg/mL (with 33.7% decrease in cell viability of HEK293 cells compared with untreated cells [19].

In contrast with these findings, after 24 h of exposure, our synthesized SnFe_2_O_4_ NPs induced cytotoxic effects against A549 lung cancer cells in concentrations higher than 100 µg/mL while inducing mild cytotoxicity on HEK293 cells at concentrations higher than 400 µg/mL in a given period. We found no cytotoxic effects for SnFe_2_O_4_ NPs in non-malignant cells derived from endothelial cells of the human umbilical vein. Future in vitro experiments using different normal and cancer cells are needed to confirm these early findings and further support the application of these newly developed SnFe_2_O_4_ NPs in theranostics.

In vivo toxicity of nanomaterials is another point of concern deserving careful investigation. In recent years, metal oxide NPs have been synthesized for medical engineering purposes, such as magnetite NPs, zinc oxide NPs, and cerium oxide NPs [20,21,22]. Despite the wide range of metal oxide NPs, there is a relative paucity of data about the potential side effects of these nanomaterials. Previous studies conducted on laboratory animals have shown the potential nephrotoxic and hepatotoxic effects of high doses of metal oxide NPs. On the other hand, some studies demonstrated that metal oxide NPs at the proper dose could have low mammalian toxicity [23]. In a previous study, the intraperitoneal administration of Xanthan gum-stabilized cerium oxide nanoparticles caused a noteworthy increment in serum antioxidant enzyme activities [24]. It seems that the time and the duration of NP administration could affect the potential toxicity of metal oxide NPs.

SnFe_2_O_4_ NPs have diverse medical, biomedical, and industrial applications. The unique physicochemical properties of SnFe_2_O_4_ NPs make them excellent candidates for medical engineering and diagnostic imaging. Previous studies conducted on cluttered cell lines have shown that SnFe_2_O_4_ NPs could induce heterogeneous Fenton reaction in the cytoplasmic reticulum, suggesting the possible cytotoxic effect of these nanoparticles on cancer cell lines [8]. In heterogeneous Fenton reaction, the Fenton catalyst converts H_2_O_2_ into free radicals that enhance free radicals in colon cancer cells. Our results showed that SnFe_2_O_4_ NPs could increase serum oxidative stress markers. It was reported that the catalase modulation of heterogeneous Fenton reaction plays a significant character in achieving selective cancer cell eradication [25].

Interestingly, there is no data regarding the biological effects of SnFe_2_O_4_ NPs on laboratory animals such as rats, mice, or rabbits. The biochemical data showed the possible nephrotoxicity of the high dose of SnFe_2_O_4_ NPs, confirmed by the histopathological investigation. The histopathological examination of testis histological sections showed no signs of reproductive toxicity. The brain histological investigation also showed no prominent toxic effects on brain tissue. These results lead us to hypothesize that SnFe_2_O_4_ NPs are not able to cross the blood-brain barrier that separates the brain from the blood [26]. Such an explanation can also be proposed for testis histology results because there is a physiological barrier in the testes, which prevents the entry of exogenous compounds, called the testis-blood barrier [27]. In the current work, the high dose of SnFe_2_O_4_ NPs caused no elevation in serum liver enzymes, while in histology investigation, some signs of slight liver damage were present. Therefore, this suggests that SnFe_2_O_4_ NPs could have hepatotoxic potential, especially at high doses or in the long-term duration.

In addition to SnFe_2_O_4_ NPs, other NP-based delivery systems were recently synthesized and evaluated using oxides or polymers for theranostics and other medical applications. The unique physical characteristics of supermagnetic NPs have made them promising tools to be used in drug delivery and diagnostic imaging. In this regard, temperature responsive core/shell Fe_3_O_4_ NPs were developed and functionalized with biocompatible copolymers [27]. Moreover, Multi-element ferrite NPs acting as heat agents were also synthesized to be used in magnetic hyperthermia treatments for breast cancer therapy [28]. Multi-anchored glycoconjugate-functionalized magnetic NPs were also synthesized for selective killing of bacteria by using an alternate magnetic field [29].

This research work provides an inclusive survey of development of tin ferrite NPs through hydrothermal method for potential application in biomedicine. Future studies will be addressed to optimize SnFe_2_O_4_ NPs for further minimizing toxicity against non-cancerous cells, and to evaluate in vivo and in vitro toxicity of SnFe_2_O_4_ NPs on different organs.

## 5. Conclusions

Magnetic NPs offer a wide variety of applications. In the current study, a hydrothermal approach is utilized for the synthesis of SnFe_2_O_4_ NPs. The synthesized NPs are analyzed exploiting FTIR, UV-Vis, XRD, and SEM techniques. The results obtained show that the SnFe_2_O_4_ are roughly spherical in shape with sizes ranging from 15 to 50 nm. The synthesized SnFe_2_O_4_ NPs have shown a favorable non-cytotoxic effect on HUVEC cells while exerting a time- and dosage-dependent cytotoxicity on human lung cancer cells. It is of great importance that these NPs did not elicit any cytotoxic effects on human umbilical vein endothelial cells. The in vivo results suggested that SnFe_2_O_4_ NPs could have a nephrotoxic potential but could be safe at low doses and, possibly, in the short term. Specifically, the in vivo experiments showed that SnFe_2_O_4_ NPs at high doses could have toxic effects on the lung, liver, and kidney of rats. In contrast, there were no effects on the brain and testis histopathology. These results suggest that SnFe_2_O_4_ NPs are not able to cross blood-brain barrier and blood-testis barrier. In order to propose the use of these NPs in theranostics, evaluating their possible impacts on human and rodent cells derived from other tissues appears warranted.

## Figures and Tables

**Table 1:** Serum biochemical parameters and lipid peroxidation in rats.

Item	Treatment			
	Control	SnFe_2_O_4_ Nanoparticles 0.1 mg/kg	SnFe_2_O_4_ Nanoparticles 1 mg/kg	SnFe_2_O_4_ Nanoparticles 10 mg/kg
MDA (nmol/mL)	39 ± 10.7	37.5 ± 8.6	35 ± 5.3	52 ± 8 *
AST (U/L)	65.6 ± 12.4	66.7 ± 15.7	77.6 ± 12.9	68.7 ± 8.8
ALT (U/L)	31.5 ± 8	37.8 ± 9.2	31.2 ± 8.6	39.1 ± 7.7
BUN (mg/dL)	11.2 ± 3.3	12.1 ± 2.6	13.2 ± 2.6	16.3 ± 4.1 *
Creatinine (mg/dL)	0.75 ± 0.15	0.81 ± 0.24	0.87 ± 0.17	1.1 ± 0.38 *

* indicates statistical significant difference with control group (P < 0.05).

## Data Availability

Data are included within this article.
